# Primary Cutaneous CD4-Positive Small/Medium-Sized Pleomorphic T-Cell Lymphoma Following Heart Transplantation

**Published:** 2017-08-01

**Authors:** B. Shakerian, N. Razavi, M. H. Mandegar

**Affiliations:** 1 Department of Cardiovascular Surgery, Shahrekord University of Medical Sciences, Shahrekord, Iran; 2 Department of Genetic, Azad University of Shahrekord, Shahrekord, Iran; 3 Department of Cardiovascular Surgery, Tehran University of Medical Sciences, Tehran, Iran

**Keywords:** Lymphoproliferative disorders, CD4-Positive T-Lymphocytes, Lymphoma, T-Cell, cutaneous, Heart transplantation

## Abstract

Post-transplantation cutaneous lymphoproliferative diseases (PTCLD) are rare, with 29 cases have so far been reported in the literature—only 4 cases underwent cardiac transplantation. Herein, we report on, to the best of our knowledge, the first case in the English literature of primary cutaneous CD4-positive small/medium-sized pleomorphic T-cell lymphoma in a cardiac transplant recipient.

## INTRODUCTION

Heart transplantation (HT) is the treatment of choice for patients with end-stage heart failure. The outcome of HT has improved dramatically over the past few decades, with increased number of patients receiving heart transplant and living with it. Post-transplant lymphoproliferative disorder (PTLD) is an increasingly recognized condition as the number of solid organ transplant recipients increases. In Iran, totally 775 patients underwent HT since 1995.

## CASE REPORT

**Figure 1:**
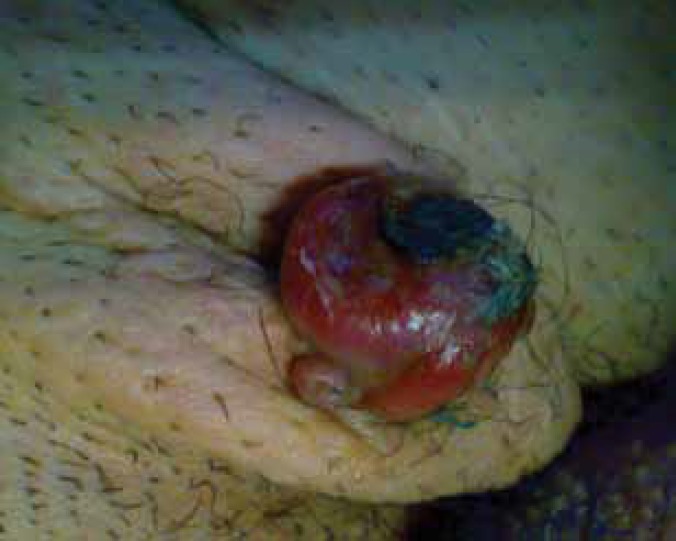
Appearance of the lesion: reddish, ulcerated, approximately 3 cm in diameter

**Figure 2:**
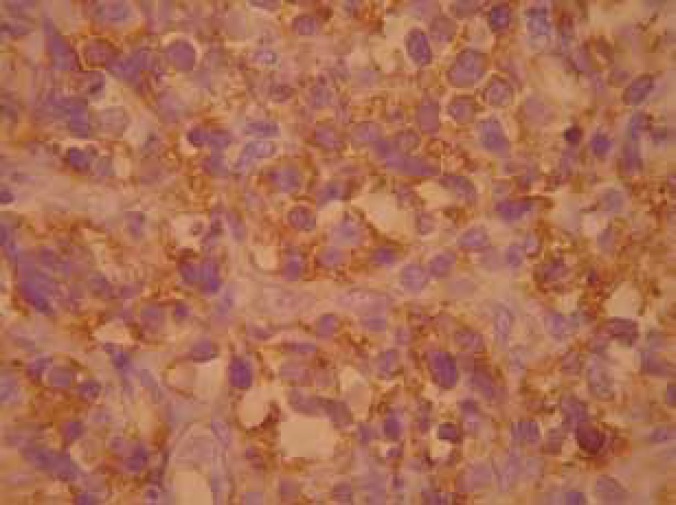
Immunohistochemical staining of the lesion: CD4^+^

In January 2011, an 18-year-old man with a history of progressive dilated cardiomyopathy underwent orthotopic heart transplantation. Standard triple-agent immunosuppressive therapy (cyclosporine, prednisolone, and azathioprine) was begun after the surgery. In May 2012, the patient developed painless left inguinal mass that expanded in size over the previous four months. Examination of the skin revealed a reddish and ulcerative mass, about 3 cm in diameter on the left inguinal area (Fig. 1). There is no inguinal, axillary, or neck lymphadenopathy. The patient underwent wide-local excision of the mass. The specimen excised revealed compact proliferation of pleomorphic small to medium sized lymphoid cells with irregular nuclear border and scanty cytoplasm. The lymphoid infiltration extended into deep dermis. Immunohistochemical (IHC) staining showed that the neoplastic cells were CD4 and CD3 positive with aberrant loss of CD5 and negative B-cell markers (CD20, CD79 α, and PUX5) in favor of post-transplantation cutaneous lymphoproliferative disease (PTCLD) (Fig 2). Examination of a peripheral blood smear was normal. Viral studies revealed no evidence of infection with Epstein-Barr virus (EBV) and Human T-cell lymphotropic virus type 1 (HTLV-1). Staging, including computed tomography of the brain, chest, abdomen, and pelvis with and without contrast, was performed and were unremarkable. Bone-marrow biopsy was normal. His cyclosporine dosage was reduced. After five years of follow-up, he was alive and doing well.

## DISCUSSION

PTLD occurs in approximately 1%–2% of transplant recipient [1]. Most cases are of B-cell lymphoma and are associated with EBV. The time between transplantation and development of PTLD is variable ranging from one month to seven years, mostly occurring within one year [2]. Estimates of PTLD frequency in recipients of different types of allografts are 1%–2% for kidney, 2% for liver, 2%–10% for heart,5%–9% for heart and lung, and 19% for intestine [3, 4]. T-cell lymphoma is more common in Asian than Western countries, usually affects adults with a higher tendency in men. Reduction of immune surveillance, chronic antigenic stimulation by the transplanted organ and direct oncogenic potential of immunosuppressive drugs could be considered risk factors for PTLD in transplant recipients. The skin is an unusual site of primary or secondary extranodal involvement of PTLD. Various types of post-transplantation primary and secondary cutaneous T-cell lymphoma, according to the European Organization for Research and Treatment (EORTC) classification, have been rarely reported in the literature. Primary cutaneous small-to-medium sized CD4^+^ pleomorphic T-cell lymphoma (PCSM-TCL) is very rare. The presentation of PCSM-TCL is heterogeneous and includes solitary papules, nodules, plaques, and tumors [5]. The prognosis is favorable, but the best treatment has not yet been defined. In conclusion, transplantation surgeons should be aware of possibility of PTCLD in cardiac transplant recipients.
